# Insights into an Optimization of *Plasmodium vivax* Sal-1 *In Vitro* Culture: The *Aotus* Primate Model

**DOI:** 10.1371/journal.pntd.0004870

**Published:** 2016-07-27

**Authors:** Kathryn Shaw-Saliba, Richard Thomson-Luque, Nicanor Obaldía, Marlon Nuñez, Sahir Dutary, Caeul Lim, Samantha Barnes, Clemens H. M. Kocken, Manoj T. Duraisingh, John H. Adams, Erica M. Pasini

**Affiliations:** 1 Department of Immunology and Infectious Diseases, Harvard T. H. Chan School of Public Health, Boston, Massachusetts, United States of America; 2 Center for Global Health & Infectious Diseases Research, Department of Global Health, College of Public Health, University of South Florida, Tampa, Florida, United States of America; 3 Center for the Evaluation of Antimalarial Drugs and Vaccines, Tropical Medicine Research / Instituto Conmemorativo Gorgas de Estudios de la Salud, Panamá, Panamá; 4 Biomedical Primate Research Centre, Rijswijk, The Netherlands; Johns Hopkins Bloomberg School of Public Health, UNITED STATES

## Abstract

Malaria is one of the most significant tropical diseases, and of the *Plasmodium* species that cause human malaria, *P*. *vivax* is the most geographically widespread. However, *P*. *vivax* remains a relatively neglected human parasite since research is typically limited to laboratories with direct access to parasite isolates from endemic field settings or from non-human primate models. This restricted research capacity is in large part due to the lack of a continuous *P*. *vivax in vitro* culture system, which has hampered the ability for experimental research needed to gain biological knowledge and develop new therapies. Consequently, efforts to establish a long-term *P*. *vivax* culture system are confounded by our poor knowledge of the preferred host cell and essential nutrients needed for *in vitro* propagation. Reliance on very heterogeneous *P*. *vivax* field isolates makes it difficult to benchmark parasite characteristics and further complicates development of a robust and reliable culture method. In an effort to eliminate parasite variability as a complication, we used a well-defined *Aotus*-adapted *P*. *vivax* Sal-1 strain to empirically evaluate different short-term *in vitro* culture conditions and compare them with previous reported attempts at *P*. *vivax in vitro* culture Most importantly, we suggest that reticulocyte enrichment methods affect invasion efficiency and we identify stabilized forms of nutrients that appear beneficial for parasite growth, indicating that *P*. *vivax* may be extremely sensitive to waste products. Leuko-depletion methods did not significantly affect parasite development. Formatting changes such as shaking and static cultures did not seem to have a major impact while; in contrast, the starting haematocrit affected both parasite invasion and growth. These results support the continued use of *Aotus*-adapted Sal-1 for development of *P*. *vivax* laboratory methods; however, further experiments are needed to optimize culture conditions to support long-term parasite development.

## Author Summary

*Plasmodium vivax* has a tremendous impact on public health; accounting for 13.8 million cases of clinical illness estimated in 2015, causing a wide spectrum of symptoms including severe disease. Development of new therapies requires a better understanding of the parasite’s biology, however, understanding the fundamental biological properties of *P*. *vivax* is challenging as there currently is no robust *in vitro* blood-stage culture system available. Unfortunately, this lack of understanding of the parasite’s basic biology, especially understanding the main variables that control blood-stage development such as nutrient-dependence, preferred host cell age, and successful *in vitro* format, is a major hurdle to the establishment of long-term *in vitro P*. *vivax* culture that is needed for basic and clinical research. In our present study, we used the *P*. *vivax* primate-adapted strain, Sal-1, obtained from *Aotus lemurinus lemurinus*, and investigated a set of the variables that control blood-stage development described in the literature. We specifically focused on addressing important parameters: host cell invasion, maturation within the invaded host cell, and egress and re-invasion into new host cells. All of which are critical for robust blood-stage growth and development.

## Introduction

*Plasmodium vivax* is the most geographically widespread human malaria parasite causing 13.8 million clinical cases every year [1]. While *P*. *vivax* was once considered the benign malaria, it can cause severe disease and death [2,3]. *P*. *vivax* presents unique challenges compared to other human malaria parasites. *P*. *vivax* forms a dormant and relapsing stage in the liver (hypnozoites) and the transmissible stages (gametocytes) are found in peripheral circulation prior to the appearance of clinical symptoms [4], making it especially difficult to interrupt transmission. Despite the large burden of disease, *P*. *vivax* has long been neglected largely due to the lack of an *in vitro* culture system for the parasite. Recent calls for worldwide malaria eradication have placed new emphasis on the importance of addressing *P*. *vivax* as a major public health problem [5].

Long-term *in vitro* culture of *P*. *vivax* has been undermined by its inability to invade mature red blood cells (RBCs), as it is restricted to immature RBCs (reticulocytes) [6], which represent as little as 0.5%-1.5% in normal blood. Reticulocytes develop in the bone marrow and are released into the peripheral circulation where they continue to mature into RBCs. Enrichment of peripheral blood have failed to support long-term *in vitro* propagation, most probably due to donor variability and age of the reticulocyte [7]. Additionally, reticulocytes rapidly mature to RBCs in culture conditions [8,9], thus requiring continual replenishment, which leads to dilution of the culture. Furthermore, besides the host cell, variability exists in the parasite as well [10]. Thus, the heterogeneity in human *P*. *vivax* patient isolates has made it difficult to establish benchmark parasite characteristics such as specific host cell age, media and *in vitro* culture formatting preferences.

Parasite growth is a product of three main variables: the ability to invade the host cell, the ability to mature within the invaded host cell and the ability to egress and re-invade new host cells. An in depth understanding the ways to successfully support each of these three parameters is necessary to address the parasites’ exponential decrease observed in *in vitro* culture conditions that has represented the major hurdle to the establishment of long-term *in vitro P*. *vivax* culture [11].

Therefore, to overcome parasite variability and establish benchmark conditions to improve the development of a robust and reproducible *in vitro P*. *vivax* culture, we set out to perform head-to-head experimental comparisons for a selected number of culture conditions using the *P*. *vivax* primate-adapted strain Sal-1 obtained from *Aotus lemurinus lemurinus*. Aware of the ethical restrictions (number of animals (3Rs principle of reduction); bleeding volume) and of the fact that *Aotus* is a scarce resource, we designed the study in such a way as to have the minimum amount of animals infected and bleedings required to obtain statistical significance in terms of biological replicas for the most important variables tested. Technical replicates (duplicates or triplicates depending on the amount of blood available) were plated for all experiments and variability was generally low between both biological and technical replicates. Due to the restrictions reported above, we had to be selective on the number of variables to include in the present study, we reviewed literature on short-term *in vitro* culture [11, 12, 13, 14 and references therein] to assess which variables appeared most critical to the development of a successful *in vitro* culture. One of the most successful attempts using an *Aotus-*adapted *P*. *vivax* strain (Chesson) was that of Golenda, et al in 1997 [14], which reported successful doubling at each generation *in vitro*. Thus, we used the apparently successful methodology described in [14] as a framework and starting point for our study.

To understand host cell requirements [11, 12, 13, 14], we tested different sources of reticulocytes and procedures for their enrichment to assess their effect on invasion efficiency. We then compared different methods of leuko-depletion to determine if there was an effect on the parasite. Furthermore, we utilized stabilized forms of nutrients to see if they could support growth. Finally, we tested different culture formatting conditions such as shaking versus static formats and altering the starting haematocrit.

## Methods

### Ethics statement

The experimental protocol was approved by the ICGES Institutional Laboratory Animal Care and Use Committee (CIUCAL) in accordance with procedures described in the “Guide for the Care and Use of Laboratory Animals,” 1996, the International Guiding Principles for Biomedical Organizations of Medical Sciences (CIOMS) and the laws of the Republic of Panama; protocol approval number 2013/06. Animals were housed at Gorgas Memorial Institute of Health Studies (ICGES) in Panama. The animals were kept in climate control rooms with 12 air changes per hour (min/max temperature set to 21/25°C and 70–80% humidity) and a red/white fluorescent 12-hour cycle starting at 03:00 pm. They were kept in pairs (male and female), in stainless steel 4 unit quads cages (Lab Products Inc., Seaford, DE, USA) with dimensions of 27 x 23.5 x 29.5 inches. Each cage was fitted with a 3⁄4-inch-diameter PVC pipe perch placed across 2/3 of the length of the cage and a 6-inch-diameter x 14.5 inches long PVC T pipe nest-box. Cages were routinely cleaned and sterilized at 180° F weekly. Diet consisted of fresh fruit, cooked potatoes, cooked meat rice, pudding (to stimulate foraging behavior and well being), and a vitamin supplement diluted in an orange juice concentrate and mixed with wheat germ bran and sugar. 3x/week animals received Monkey Chow (New World Primate Diet 5040, LabDiet; PMI Nutrition International, LLC, Brenhwood, MO, USA) and fresh fruit. Water was administered daily in plastic bottles fitted with a zip tube (Girton; Millville, PA, USA). During routine bleeding procedures the animals were sedated with Ketamine at a dosage of 10 mg/Kg IM. No euthanasia was carried out during these experiments and once the animals were radically cured of malaria with mefloquine at 20 mg/kg orally once, they were transferred to the reproductive colony. All animals received daily veterinary care.

### Animals and parasites

For the donor inoculum, female laboratory-bred and spleen intact *Aotus lemurinus lemurinus* monkeys, weighing between 789–850 g were infected with *Aotus*-adapted *P*. *vivax* Sal-1 and parasites were obtained after bleeding at a majority of ring stages. Parasites were cryopreserved with Glycerolyte (Baxter) following the Methods in Malaria Research [15]. The animals were housed at Gorgas Memorial Institute of Health Studies in Panama City, Republic of Panama and cared and maintained as described [16], in accordance with the reviewed and approved protocol entitled “Production of *Aotus Plasmodium vivax* SAL-1 and AMRU-1 infected blood for continuous *in vitro* culture attempts” submitted in 2013, registered in the ICGES-Institutional Animal Care and use Committee (CIUCAL-ICGES) under the accession number 2013/06 and following the criteria set forth in the International Guiding Principles for Biomedical Organizations of Medical Sciences (CIOMS) and the laws of the Republic of Panama.

**Fig 1:**
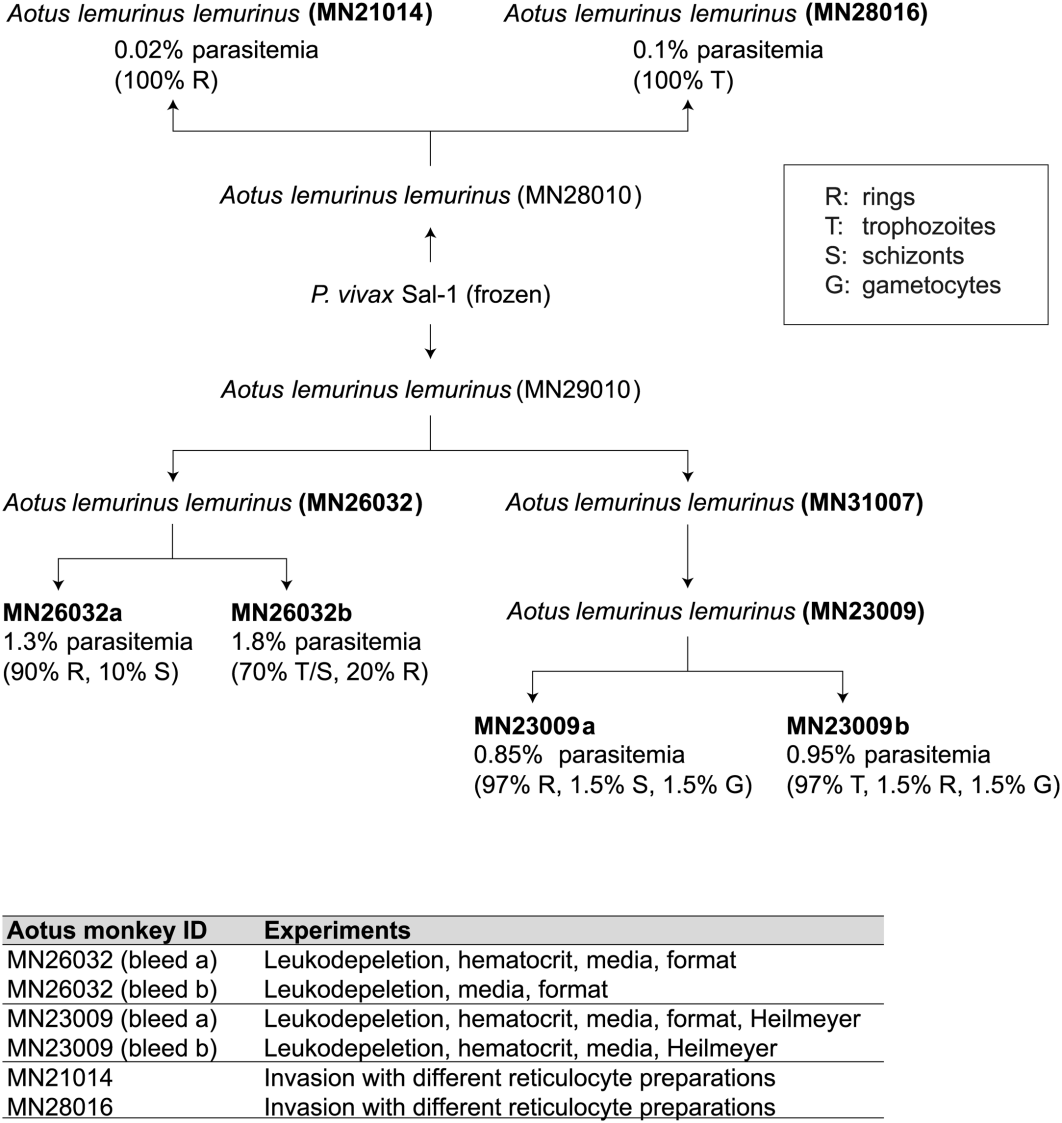
Experimental design using *Aotus lemurinus lemurinus* monkeys. Diagram showing the inoculations of the *Aotus* monkeys with *P*. *vivax* Sal-1. The percentages shown are for staging of the parasites at bleed (R, rings; T, trophozoites; S, schizonts; G, gametocytes). Table shows the experiments that each bleed was used for.

Experimental monkeys were infected with parasites through the saphenous vein. Each parasite inoculum contained 5.4x10^6^ *P*. *vivax* Sal-1*-*parasitized erythrocytes in a volume of 1 mL. Parasitemia was monitored daily following the Earle and Perez method [17] with Giemsa (Sigma) stained thick blood smears using 5 μL of blood collected by gently pricking the outer ear vein of the animals. Once the parasitemia had reached a peak, the different animals were bled. A total of 3mL (1–1.5 mL packed cells) was collected. To maximize the number of biological replicates while still limiting the number of animals we infected, we bled some monkeys twice (MN23026 and MN23009) but we took less blood at each draw so the total amount of blood collected was 3 mL. Immediately, following bleeding, the animals were radically cured with mefloquine orally at 20 mg/kg once as described [16]. At each bleeding thin Giemsa blood smears were prepared to obtain precise parasite counts and perform asexual (rings, trophozoites and schizonts) and sexual (gametocytes) staging before parasites were put in culture to test different culture conditions as described throughout the manuscript. The schematic describing the passaging is in Fig 1.

### Heilmeyer classification of *ex vivo* and *in vitro* parasites

**Fig 2:**
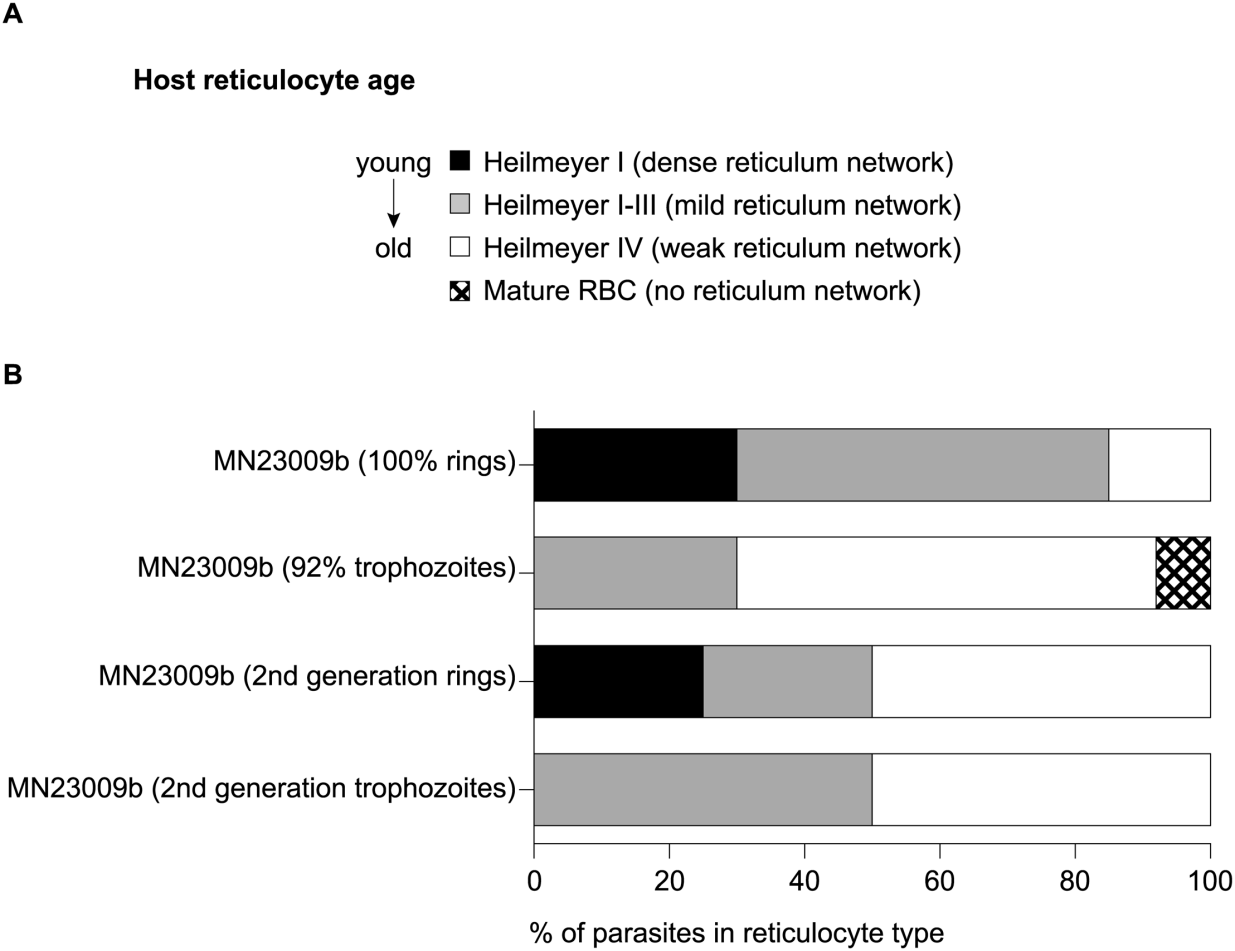
Co-staining of reticulocytes and parasites reveals *ex vivo P*. *vivax* Sal-1 is found in reticulocytes of different maturity. (**A**) Heilmeyer classification according to [19.22]. The youngest reticulocytes will have a dense reticulum network when stained with NMB (Heilmeyer I), older reticulocytes will have a mild reticulum network (Heilmeyer II-III) and the oldest reticulocytes will have weak reticulum network (Heilmeyer IV). Mature RBCs will not stain with NMB. (**B**) Two independent biological replicates, MN23009a and MN23009b were examined for the Heilmeyer classification of the host reticulocyte infected with *P*. *vivax* Sal-1. Smears were stained first with NMB for the reticule network, followed by Giemsa staining for the parasite’s nucleus and ribosomes. MN23009a was drawn at 100% rings while MN23009b was drawn at mostly trophozoites. For MN23009b, parasites were kept in culture for 20 hr and the resulting second-generation rings and trophozoites were assessed for host cell Heilmeyer classification. S1 Fig contains examples.

To evaluate the age of the reticulocytes that contained parasites, we used a double staining method, which simultaneously stains the reticulocyte and the parasite. 5 μL of packed infected cells were washed with PBS, mixed with an equal volume of new methylene blue (NMB) (Sigma) and allowed to stain for 15 minutes. Following the staining, the cells were smeared on a glass slide (Falcon) and air-dried. Slides were then methanol fixed and stained with Giemsa. In the resulting slides both the reticulum of the reticulocytes and the nuclear and cytoplasmic material of the parasites were stained (see S1 Fig, Fig 2).

### Preparation of reticulocytes for invasion

Fresh reticulocyte preparations were obtained from Duffy antigen positive hemochromatosis blood (Brigham and Women’s Hospital and The German Red Cross Blood Bank) and Buffy packs (The Interstate Blood Bank INC. Memphis, USA). Three reticulocyte enrichment procedures were used: (i) a modified differential centrifugation method used in [14] (ii) a Percoll gradient [18] and (iii) a CD71^+^ immuno-magnetic purification method (Miltenyi, manufacturer’s specification).

**Fig 3:**
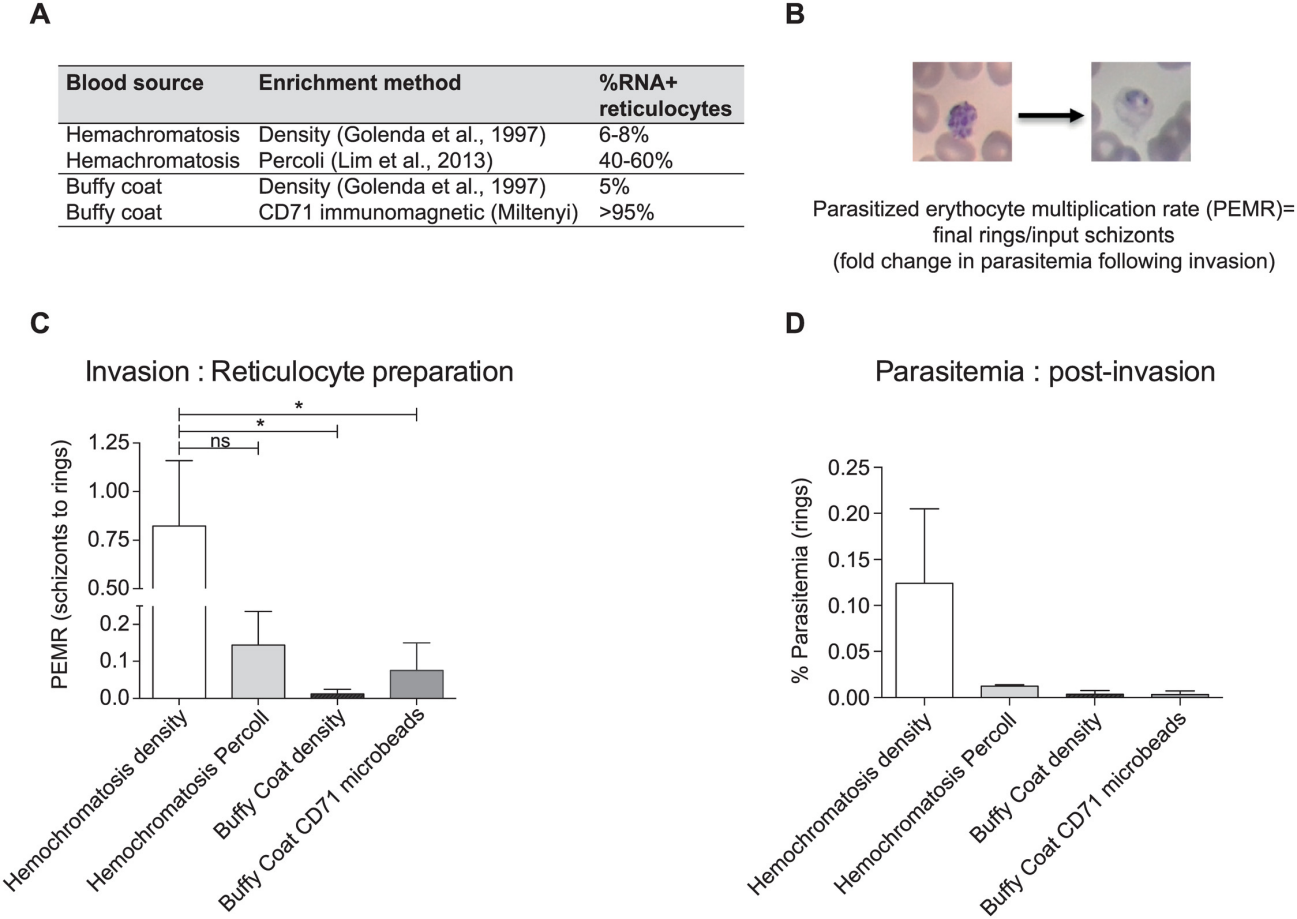
Reticulocyte enrichment method can affect invasion of *P*. *vivax* Sal-1. (**A**) Table showing methods used to enrich reticulocytes from peripheral blood derived either from hemochromatosis patients and Buffy Coat blood packs. The enrichment was performed according to either published studies or manufacturer’s instructions detailed in the Materials and Methods. The resulting total reticulocyte percentage was determined by staining with NMB. (**B**) The final fold change in parasitemia following invasion can be normalized to compare different invasion assays using the parasitized erythrocyte multiplication rate (PEMR) by dividing the final number of rings by the input number of schizonts [18]. (**C**) PEMR demonstrates that the hemochromatosis reticulocytes enriched by density using the [14] method resulted in the best invasion in two independent biological replicates. This invasion was better than the invasion in the same blood enriched by Percoll and significantly better than the reticulocytes from Buffy Coat enriched by density or CD71-microbeads. Parasites were from *Aotus* MN21014 and MN28016. Statistical significance was determined using Dunnett’s multiple comparison test. P value <0.05 (**D**) Final ring parasitemias of the invasion assays in B. Error bars represent the standard error.

1. Differential centrifugation was carried out as follows: white cells were removed using a leukocyte separation filter (Sepacell), centrifuged (1,000 *g*, 15 min, 22–24°C), and excess plasma was removed from each tube to achieve an 80–85% hematocrit (hct) and stored 1 hour at 37°C. Warmed blood was then centrifuged (3,200 *g*, 30 min, 32°C) and the plasma aspirated to approximately 6 mm above the top of the pellet. Remaining plasma from the previous centrifugation (kept at 4°C) and the upper 25% of the pellet were pooled to a 50% HCT and stored at 37°C. This suspension was then diluted with an equal volume of fresh, homologous plasma (50% HCT) and the 37°C incubation, centrifugation, and aspiration procedures were repeated except that only the top 10% of the final pellets were collected and pooled. Reticulocyte purity was assessed with NMB staining.
2. Percoll gradients were performed according to Lim et al [18] by adjusting the hemochromatosis blood to a 50% haematocrit using McCoy’s 5A medium, and layered on a 70% Isotonic Percoll cushion and centrifuged for 15 min at 1200 x g, A band of concentrated reticulocytes was formed at the Percoll interface after centrifugation for 15 min at 1200 x g. Reticulocytes were removed, washed in McCoy’s 5A medium and stored at 4°C. Reticulocyte purity was assessed with NMB staining.
3. CD71^+^ immuno-magnetic purification was performed according to manufacturer’s (Miltenyi Biotech) specifications. Briefly, 5mL of previously washed Buffy coat pellet, was filtered on a NEO Leukocyte Reduction filter and then mixed with 10mL cold autoMACS running buffer (Miltenyi Biotech) and incubated with 200μL human CD71 microbeads (Miltenyi Biotech) in a 50mL centrifuge tube for 30min at 4°C in an oscillator. After washing the cells with 50mL autoMACS running buffer, they were re-suspended in the same buffer and placed in a 50mL pre-cooled rack for CD71+ enrichment on an autoMACS Pro Separator in the positive selection mode (Posselds). CD71+ eluted cells were washed in the same buffer twice and New Methylene Blue thin blood smears were performed after a 1:1 dilution and 15min incubation at 37°C to check for purity, yielding normally >90% (Fig 3).

Frozen enriched reticulocytes preparations were obtained from Duffy antigen positive hemochromatosis blood collected by the German Red Cross Blood bank using either (i) differential centrifugation or (ii) a Percoll gradient as described above. After enrichment reticulocytes were frozen with homologous plasma and 10% DMSO in cryotubes. Except for the frozen cells, reticulocytes were enriched and stored at 4°C and used within one week of preparation.

### Leukocyte-depletion methods for *P*. *vivax*-infected *Aotus* blood

**Fig 4:**
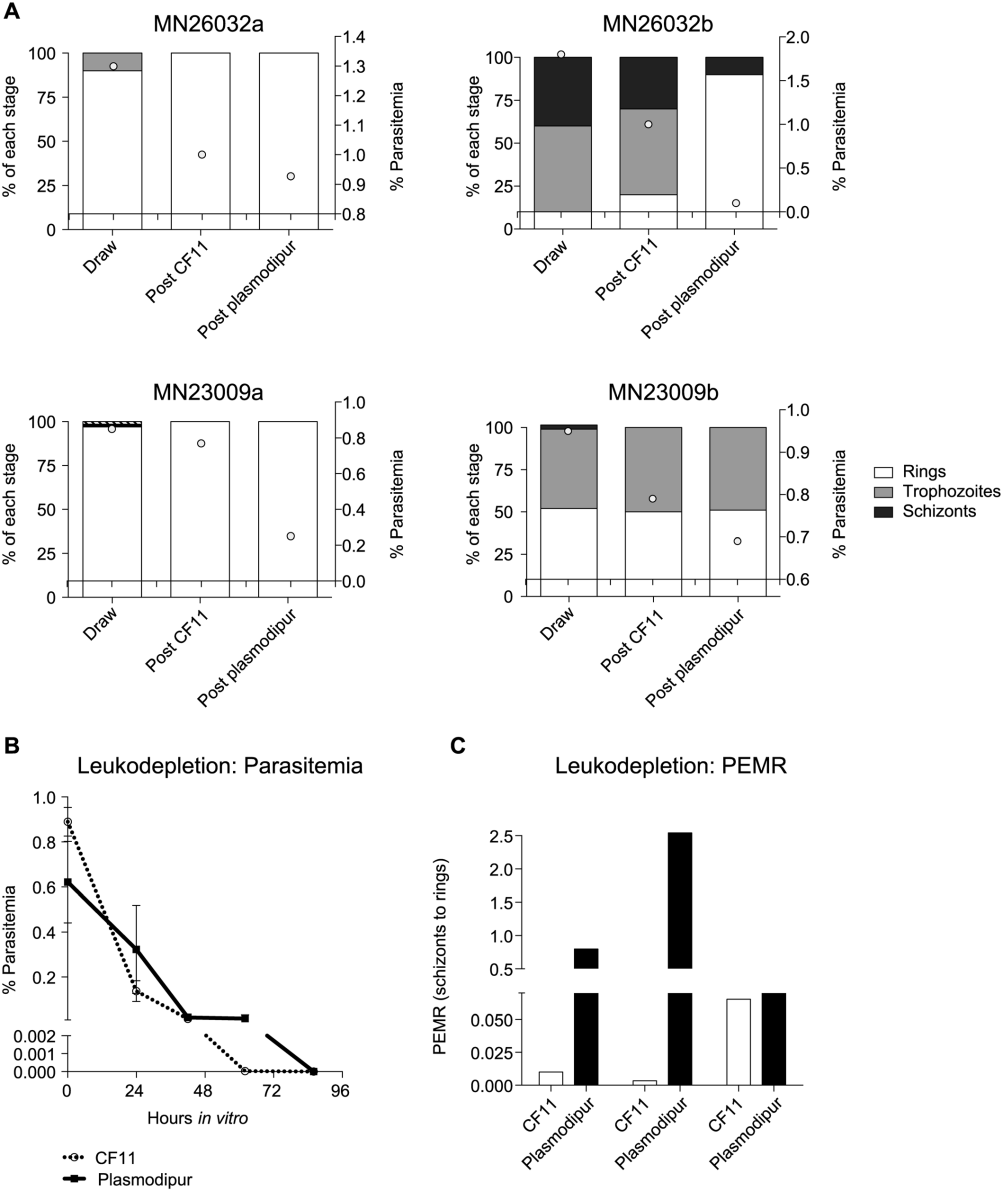
Leukodepeletion can affect *P*. *vivax* Sal-1 parasites from *Aotus*. (**A**) Staging and parasitemia levels for four independent biological replicates from two *Aotus* monkeys. Decrease in parasitemia levels occurred with later stage parasites after Plasmodipur filtration compared to CF11 filtration in MN26032a (enriched in rings), MN26032b (mixed stages), MN23009a (enriched in rings) and MN23009b (mixed stages). (**B**). Parasites from the four independent biological replicates had similar parasitemias throughout their time *in vitro*. (**C**) However, differences were observed in the PEMR in parasites from CF11 and Plasmodipur. This was mostly contributed by one biological replicate, MN23009a and the trend was not statically significant (Mann Whitney). Error bars represent the standard error.

Two different leuko-depletion methods were compared: (i) CF11 columns and (ii) Plasmodipur filters. For all experiments, Giemsa stained smears were made before and after leuko-depletion to assess parasitemia and stage and health of the parasites. Parasitemia was determined by counting the number of parasites per 10,000–20,000 RBCs per slide as presented in Fig 4.

1. CF11 (Whatman cellulose) columns were prepared as follows: the plunger on a 10 mL syringe (BD biosciences) was removed and a cotton plug was added. CF11 powder was loosely packed to ~6–7 mL on the syringe. Glass beads (Sigma) were added to the top to flatten the CF11. The column was prewashed with 3% BSA/PBS. *P*. *vivax-*infected *Aotus* blood was spun-down and the plasma was removed. Blood was re-suspended to ~50% hct with 3% BSA/PBS and run through the column. The eluted blood was collected and the column was washed with 3% BSA/PBS until the column ran clear. The blood was then centrifuged and the supernatant was removed.
2. Standard Plasmodipur filter units (Gentaur Gen218839) were connected to a plastic 10 mL syringe and washed with McCoy’s 5A medium. Blood was spun-down at the plasma was removed. The blood was mixed with an equal volume of PBS and poured into the barrel of the 10 mL syringe that is connected to the Plasmodipur filter. The plunger was gently inserted into the syringe barrel and the blood was filtered drop wise into a 50 mL tube. The filter was washed with an additional 10 mL of PBS and the wash collected into the 50 ml tube.

### Measurements of growth (conversion) and invasion (parasitized erythrocyte multiplication rate, PEMR)

Throughout the study, we used two methods to standardize and normalize our measurements so we could compare different biological replicates. At each time-point, slides were made and Giemsa-smeared to evaluate parasitemia, stage and health of the parasites. As parasites were drawn at roughly synchronous stages (e.g. mostly rings or mostly trophozoites) and progressed *in vitro* synchronously, we were able to calculate conversions between different stages. Conversion was determined by staging the parasites at each time-point and multiplying the percent of each stage by the parasitemia (initial rings or trophozoites). The numbers of parasites were counted and percentages were calculated in the same way (resulting trophozoites or schizonts). The percent conversion was the calculated by taking the resulting parasitemia divided by the initial parasitemia. For invasion, we used a normalized measurement of the final ring parasitemia called parasitized erythrocyte multiplication rate (PEMR) [18]. Here, the final number of rings is divided by the input number of schizonts. Our PEMR calculations include only healthy rings with intact nuclei and ring cytoplasm on smears and do not include pyknotic cells.

### General culture conditions

As Golenda et al, 1997 was the only study to see doubling over multiple generations, we followed the formatting and media conditions that were used [14]. Therefore, unless specified, for all experiments, we cultured parasites in 4-well plates from Nunclon (Sigma D6789-1CS, Nunc176740) at 6% haematocrit (30μL of iRBCs in 500 μL of media). Unless otherwise stated, media for all experiments was prepared as follows: McCoy’s 5A Modified medium (Gibco) was supplemented with 25 mM HEPES (Sigma), 2 g/mL sodium bicarbonate (Sigma), 2 g/L D-glucose (Sigma) and 40 μg/mL gentamycin (Sigma). Media was supplemented always with 20% AB+ heat inactivated serum (The Interstate Blood Bank, Memphis, TN, USA). A candle jar was used to maintain the gas conditions of the 4-well plates (~18% O2). Cultures were maintained at 37°C. For invasion, once the majority of parasites reached the schizont stage, uninfected, human density-enriched reticulocytes were added at a 1:1 ratio doubling the haematocrit to 12% and after re-invasion cultures were diluted back to 6% haematocrit [14].

### Special culture conditions

**Fig 5:**
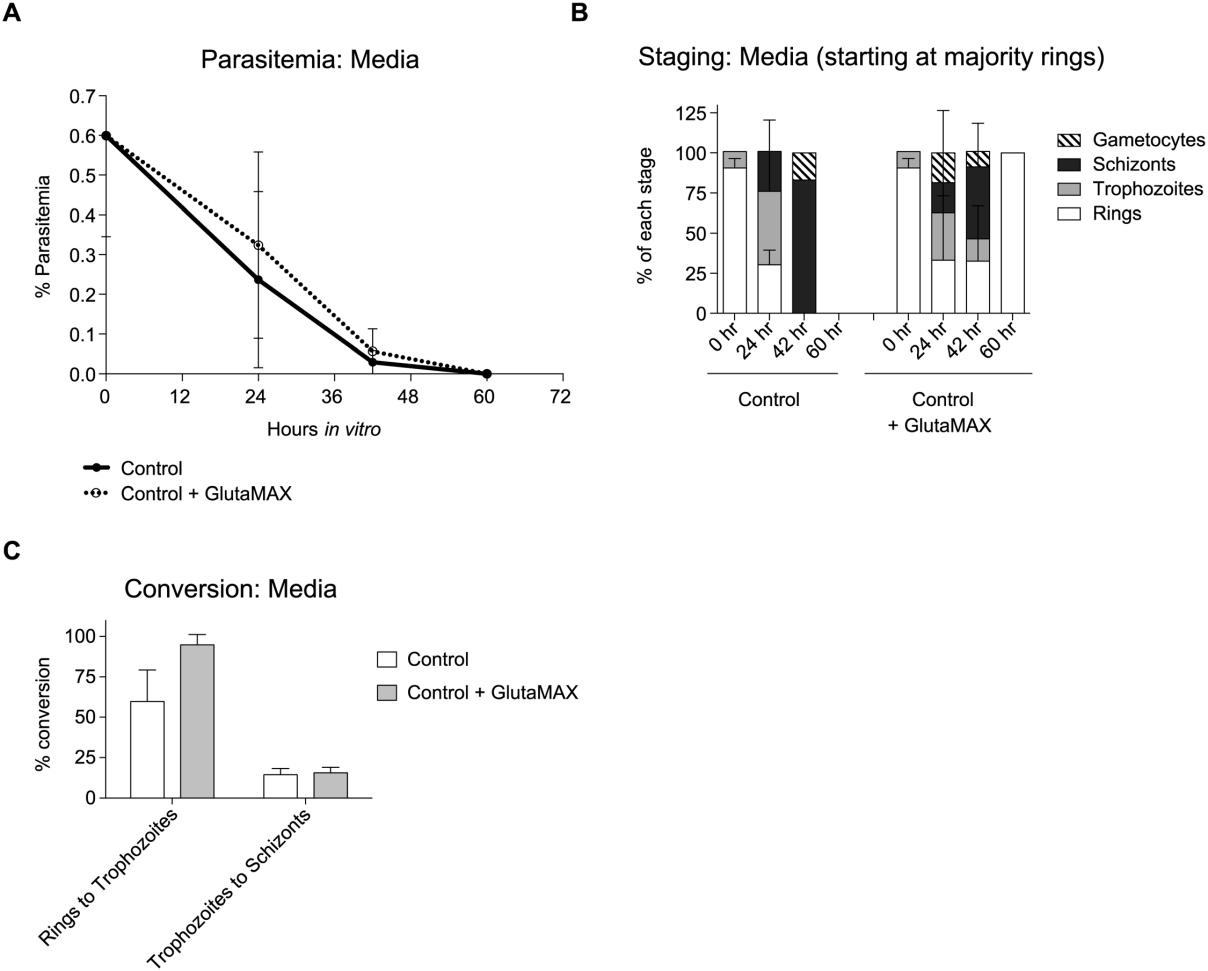
Media supplementation can influence the health and growth of the parasite. (**A**) Parasitemia graph showing the effect of GlutaMAX on parasitemia at different time points using three independent biologicals *P*. *vivax* Sal-1 parasites from MN23062a and b and MN23009a. Reticulocytes from hemochromatosis patients enriched by density were used for these experiments. (**B**) Histogram comparing parasite stages at different time points from the parasites in A: (**C**) Conversion percentages of rings to trophozoites and trophozoites to schizonts in media supplemented with or without GlutaMAX. Data from the three independent biological replicates in A. Error bars represent the standard error. While the parasitemia differences are not statically significant, we did observed a longer persistence of parasites in the cultures supplemented with GlutaMAX and we did observed reinvasion only in the GlutaMAX culture.

To determine the effect of stabilized nutrient components, 100X GlutaMAX (Gibco) was supplemented to a final 1X (Fig 5).

**Fig 6:**
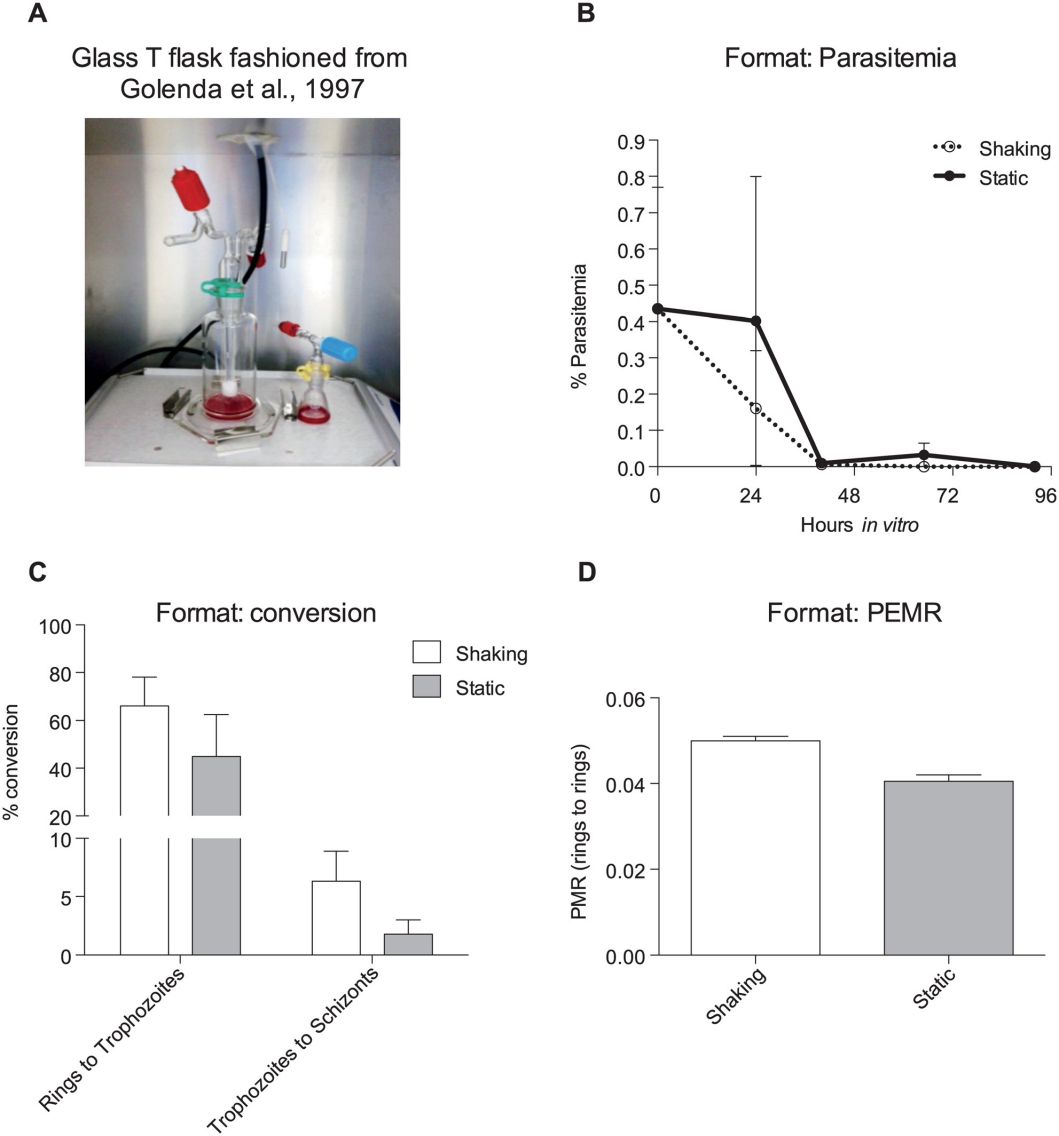
Shaking and static format support cultures similarly. (**A**) Glass T flask used for shaking conditions to replicate the conditions used in [14]. (**B**) Parasitemias of two biological replicates (MN23026b and MN23009b) grown in shaking or static conditions at equal haematocrits. (**C**) Conversion rates between rings to trophozoites or trophozoites to schizonts in the two biological replicates (**D**) Parasitized erythrocyte multiplication rate (PEMR) of the two biological replicates in either shaking or static conditions using reticulocytes from hemochromatosis patients enriched by density. Error bars represent the standard error. Results are not statically significant.

For the cultures in which we wanted to compare shaking versus static culturing formats, we had glass flasks specially made to the specifications of the T flasks that were used in 1997 [14]. Customized glass Erlenmeyer 5 mL and 10 mL funnels and type T funnel fuzzier cock and sintered Type T culture cylinder were manufactured by Ritmester B.V (Fig 6A). Flasks were gassed using standard parasite gas mixture (5%O2, 5%CO2, 90%N2) by attaching a specialized tube to the fuzzier cock. Shaking was done at 100 rpm on an orbital shaker (Thermo). Although with different volumes of media, haematocrit was respected to a 6%. These conditions mostly closely replicate [14].

**Fig 7:**
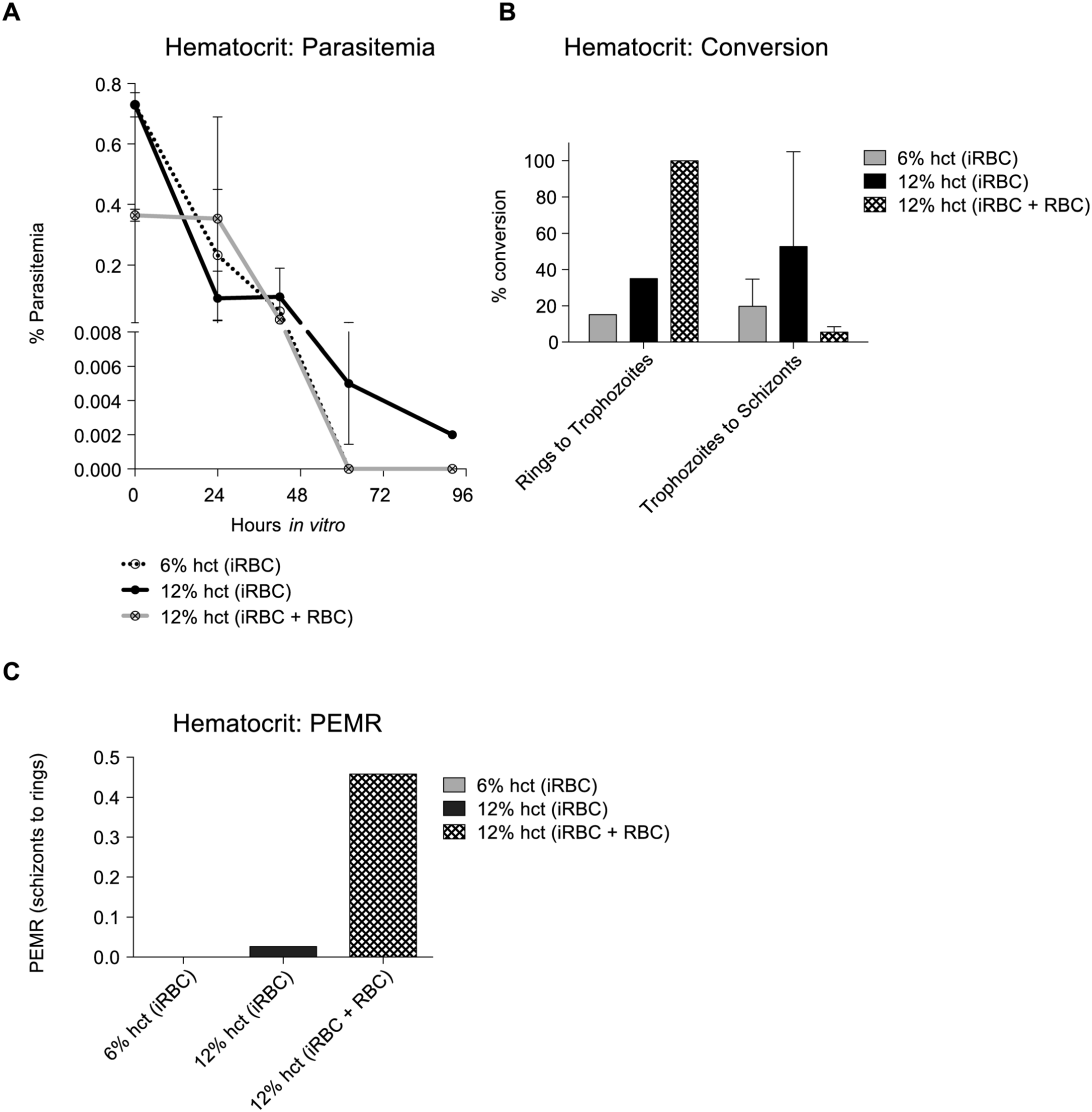
Starting haematocrit (hct) can influence *in vitro* outcome of *P*.*vivax* Sal-1. Cultures from two independent biological replicates, MN23009a and MN23009b, were initiated at different hcts. Reticulocytes enriched by density from patients with hemochromatosis were used. Cultures were initiated with the initial draw at 6% or 12% hct (iRBC, infected RBC) or were initiated with the 6% initial draw (iRBC) and hct was doubled to 12% hct with uninfected enriched reticulocytes (density hemochromatosis) (12% iRBC + RBC) to account for any differences due to the overall total parasite biomass. (**A**). Parasitemia over time from the two biological replicates. Survival was affected by the conversion differences in B as the two cultures were initiated at majority rings (MN23009a) or mixed rings and trophozoites (MN23009b) (**B**) Conversion rate differences in the two biological replicates in A between rings to trophozoites and trophozoites to schizonts. (**C**) Parasitized erythrocyte multiplication rate was only possible to measure in one biological replicate, MN23009a. Error bars represent the standard error. To set up a culture at 12% hct, we first set up the culture at 6% hct and doubled the hct with uninfected fresh, hemochromatosis human blood (12% iRBC + RBC). Results were not statically significant.

When culture outcomes where studied regarding the starting hct, this was varied from standard 6% to 12% and 15%. Cultures were initiated at the indicated hcts using the blood directly from the draw and diluted to the appropriate hct with complete media (iRBC). We also tested a culture at 12% hct that was mixed 6% from the draw and doubled with uninfected blood (iRBC + RBC) (Fig 7).

### Statistical analysis

Statistical analyses were performed using Graph Pad Prism (5.1).

## Results

### *Aotus lemurinus lemurinus* and *P*. *vivax* Sal-1

Four adult *Aotus lemurinus lemurinus* monkeys (MN21014, MN28016, MN26032 and MN23009) served as donors of *P*. *vivax*-infected reticulocytes for this experiment after being sub-passaged from MN28010, MN29010 and MN31007 (Fig 1). To maximize the number of biological replicates for the media and formatting experiments, we bled MN26032 and MN23009 twice. While this limited the amount of starting blood for each condition, it maximized the replicates without having to infect additional animals. Their parasitemia was monitored daily by Giemsa stained thick blood smear. For the reticulocyte preference and invasion assays, we needed larger amounts of blood so we only bled MN21014 and MN28016 once (3mL) to maximize the blood volume. Here we are using the same batch of frozen parasite stocks for all monkey infections, as conventionally done in monkey ex-vivo drug studies, where generally infected blood from one single animal is used. When planning these experiments, we attempted to be as thorough as possible while keeping sight of the important ethical boundaries established for the work with non-human primates (3Rs principle of reduction) and of the fact that *Aotus* is a scarce resource. We have designed the study in such a way as to use the minimum amount of animals/bleedings required to obtain statistical significance in terms of biological replicas for the most important variables tested. Technical replicates (duplicates or triplicates depending on the amount of blood available) were plated for all experiments; variability was generally low.

Careful parasite staging was carried out on all smears as a way to strictly characterize the starting population of *P*. *vivax*-infected reticulocytes. A careful staging clearly demonstrates that the synchronicity of the parasite populations differs between monkeys and between blood draws and that it is therefore important to refer each dataset to its original population when invasion or maturation and growth are considered. A table is provided detailing the experiments each bleed (biological replicate) was used for (Fig 1) and the results from experiments where we had biological replicates are reported in the main text. Due to the fact that *Aotus* is scarce resource and widely accepted ethical constraints apply to this research, the number of animals was restricted to the minimum required to have enough biological replicates to test a selected number of variables. As we sometimes had small amounts of leftover infected blood, we considered it unethical to discard any material and we therefore used the excess to look into other variables of interest identified in the literature [11, 12, 13, 14], the results of which are shown in Supplemental Materials.

### *Ex vivo P*. *vivax* Sal-1 is found in reticulocytes of different ages

To identify the age of reticulocytes that best supports *P*. *vivax* invasion and growth, we surveyed the parasites coming directly from the bleed (*ex vivo*). Heilmeyer classification [19] based on New Methylene Blue (NMB) staining can be used to determine the age of the reticulocyte [10]. In four biological replicates, we found the youngest reticulocytes contain large amounts of RNA (reticulum) and stain most intensely (Heilmeyer I) while old reticulocytes will have very week staining (Heilmeyer IV) (Fig 2A and S1 Fig). Mature RBCs do not contain RNA and will not stain with NMB (no Heilmeyer classification). Double-staining with NMB and Giemsa revealed that ring stage samples isolated directly from *Aotus* showed 30% of the parasites being hosted inside strongly NMB stained cells with a very dense reticulum network (Heilmeyer I), 55% inside weakly NMB stained cells with a mild reticulum network (Heilmeyer II-III) and only 15% of the host cells presented either a weak reticulum (Heilmeyer IV) (Fig 2B and S1 Fig). Although further experiments would be required to demonstrate this finding, it suggests that the parasites preferentially invade younger reticulocytes *in vivo* in the *Aotus* model. When more mature samples predominantly containing trophozoites were obtained directly from the *Aotus*, the 30% of the parasites were still found in the strongly stained Heilmeyer II-III while the rest of the parasites were found in Heilmeyer IV reticulocytes (62%) and only 8% of the parasites were found in cells lacking NMB (mature RBCs) (Fig 2B and S1 Fig), indicating that if merozoites preferentially invade young cells, reticulocyte maturation of *P*. *vivax* infected cells progresses *in vivo*. Interestingly, *in vitro* data followed a somewhat different pattern where second-generation newly invaded rings (20 hr *in vitro*) were found 25% inside Heilmeyer I, 25% in Heilmeyer II-III and 50% in Heilmeyer IV (Fig 2B). When second generation trophozoites (20 hr in vitro from a more mature sample) were studied: 50% were inside Heilmeyer II-III and 50% in Heilmeyer IV. Whether this is due to reticulocytes maturing *in vitro* prior to parasite invasion or the parasite driving accelerated maturation of the reticulocyte *in vitro* is unclear.

### Reticulocyte enrichment methods affect invasion efficiency

Given the findings of the *ex vivo* staining and a recent report indicating reticulocyte preparation method can affect the success of invasion [20], we sought to understand if the reticulocyte source (blood from routine phlebotomies or Buffy packs) and enrichment method affected parasite invasion. In this experiment we performed a head-to-head comparison of invasion rates with reticulocytes from different sources enriched following the different procedures presented in the Material and Methods (density centrifugation, Percoll enrichment, immunomagnetic separation (Fig 3A) and frozen versus fresh reticulocytes (S2)). In two independent biological replicates (MN21014 and MN28016) and two separate experiments with technical replicates, we calculated the normalized invasion by PEMR (Materials and Methods, Fig 3B). Dunnett’s multiple comparison showed that *P*. *vivax* demonstrated a significant preference for fresh hemochromatosis blood derived reticulocytes enriched by differential centrifugation (Fig 3C and 3D), despite the fact that the reticulocyte numbers were lower than other methods of preparation (Fig 3A). In order to verify that human reticulocytes that were invaded as opposed to back invasion into the endogenous *Aotus* reticulocytes present from the original inoculum remaining in the culture, we performed flow cytometry analysis to determine the amount of *P*. *vivax* (PyBIP Alexa647) inside human cells (Glycophorin A/CD235A+) versus *Aotus* cells (Glycophorin A/CD235A-) (S2A and S2B Fig). BIP is an endoplasmic reticulum (ER)-targeted protein binding immunoglobulin protein whose *P*. *falciparum* version (PfBIP) reactivity against *P*. *vivax* has already been demonstrated [21].

We also tried reticulocytes from different hemochromatosis donors in Europe that had been enriched by the same methods as the fresh hemochromatosis blood but had been cryopreserved to preserve freshness in transit. Limited numbers of cells were recovered post-thaw so only one parasite biological replicate was tested (S2 Fig). Again, fresh, density enriched blood proved to provide a higher PEMR and resulting ring parasitemia (S2C and S2D Fig).

These results indicate that cryopreservation and Percoll may damage or somehow intrinsically alter reticulocytes during enrichment and preservation, thereby preventing successful invasion of the parasite. Therefore, in subsequent experiments, we decided to restrict ourselves to fresh hemochromatosis reticulocytes enriched by differential centrifugation to monitor invasion, growth and egress.

### Leuko-depletion methods can retain late stage parasites and affect invasion efficiencies

[14] used CF11 to deplete leukocytes. However, CF11 is no longer manufactured and most laboratories now use commercially purchased Plasmodipur filters or cellulose powder. Therefore, we set out to compare the affect that these two leuko-depletion methods have on parasitemia, staging and health of the parasite. In a few cases we found a correlation between the stages observed in the blood draw, the filtration procedure used and the course of parasitemia. In cases of mixed stage blood draws involving majority mature (trophozoite and schizont stage) parasites, the parasitemias post-Plasmodipur filtering was lower (sometimes, markedly so) when compared to post-CF11 parasitemias (Fig 4A). In four biological replicates, no major differences were observed in parasitemia over time, but the Plasmdipur-filtered parasites did persist for longer in one replicate (Fig 4B). Interestingly, in the four biological replicates, we found that the CF11-filtered parasites appeared compromised during invasion as indicated by a lower PEMR (Fig 4C). Although the trend was not significantly significant, as most of the observed difference was mostly contributed by a single biological replicate, MN23009a. Together these findings indicate that CF11 filtration retains less of the mature stages compared to Plasmodipur, but the mature stages present in CF11 elution may be less fit. This mixed outcome may be due to the contact with the CF-11 powder or due to the fact that CF11 allows *ex vivo* late stages to pass through, which may be less amenable to adapt to *in vitro* culture conditions than the early *ex vivo* stages.

### Supplementation of media can influence growth

We tested various preparations of media and supplements. While the parasitemias were not statically different (Fig 5A), a stabilized form of L-glutamine, GlutaMAX (Gibco), consistently supported parasite maturation along the whole parasite cycle in four independent biological replicates (Fig 5B). Greater benefits were observed in the transition from ring to trophozoite, with better conversion rates compared with McCoy’s 5A modified medium (control) alone (Fig 5C). Conversion from trophozoites to schizonts were somewhat negligible (Fig 5C), but overall, both parasites and host cells appeared healthier in the media supplemented with Glutamax as evidenced by reinvasion of schizonts in the supplemented cultures compared to the control media alone (Fig 5B). GlutaMAX is a dipeptide of L-alanyl-L-glutamine, which does not break down to form toxic byproducts such as ammonia like traditional L-glutamine. These results indicate that *P*. *vivax* could be acutely sensitive to build-up of waste or toxic products in *in vitro* conditions.

### Shaking does not enhance invasion and has marginal influence on development

An important contradictory finding in the literature is the effect of shaking versus static conditions on *in vitro P*. *vivax* development. Golenda et al. [14] reported that shaking was necessary to facilitate egress and reinvasion while Mons et al. [12] reported that this actually harmed the parasites. To determine the effect of shaking, we compared both invasion and development of the parasites in shaking or static cultures. To exactly recapitulate the findings of Golenda et al. [14], we had special T type glass flasks constructed (Fig 6A). We found that the parasitemia remained higher and parasites persisted in the culture for longer when the cultures were maintained in static conditions (Fig 6B), similar to what Mons et al. reported [12]. In fact, while shaking and static cultures had similar conversion rates from rings to trophozoites (Fig 6C), shaking appeared to be somewhat only marginally beneficial to conversion from trophozoites to schizonts (Fig 6C). Despite Golenda’s findings, we determined that shaking had a negligible effect on invasion; PEMR 0.05 compared to static PEMR of 0.04 (Fig 6D). None of these results were statically significant.

### Haematocrit (hct)

In one biological replicate, MN26032a, we initiated cultures at 6% hct and 15% hct to determine if there was any effect on parasitemia (in static cultures). Interestingly, we observed a higher parasitemia that persisted for longer *in vitro* (S3 Fig). To examine this further, we decided to test two biological replicates, MN23009a and MN23009b, at different hcts at the initiation of the culture. We started the culture at either 6% hct or 12% hct (iRBC). To determine if the findings from S3 Fig (MN26032) were due to a higher overall parasite biomass, we also set up a culture at 12% hct but this time, we set up the culture at 6% hct and doubled the hct with uninfected blood (12% iRBC + RBC). Overall, we found that the 12% hct culture had a higher parasitemia overall, although no statically significant (Fig 7A). Interestingly, the parasites persisted for longer *in vitro* in the 12% hct condition (Fig 7A). Interestingly, the 12% hct iRBC + RBC provided the best conversion from rings to trophozoites but the 12% hct iRBC had the best conversion from trophozoites to schizonts (Fig 7B). Additionally, the 12% hct iRBC + RBC had a higher PEMR overall (Fig 7C). While these results are not statically significant, the trends indicate that differences in parasite biomass and formatting changes to the hct could influence the outcome of the culture.

## Discussion

Since Bass and Johnson’s first attempt to culture *P*. *vivax* in 1912 [22], many more unsuccessful attempts have been made to develop a continuous, long-term *in vitro* culture of *P*. *vivax*. To date, the most successful of such attempts has been the one by Golenda et al. [14] in 1997 in which they were able to maintain *P*. *vivax* in culture for 8 cycles, doubling the number of parasites at almost every cycle and thus maintaining a reasonably high parasitemia compared to other reports [23, 24]. Equally important the Golenda study [14] confirmed that non-human primate-adapted *P*. *vivax* (Chesson) derived from infections carried out in *Aotus nancymai* and *Aotus lemirinus griseimenbra* could be successfully adapted to *in vitro* culture conditions. The scope of our experiments was to use this one successful attempt and to optimize culture conditions for the *Aotus lemurinus lemurinus* adapted *P*. *vivax* Sal-1. These studies were designed to inform and improve standardized conditions for further testing of an *in vitro* culture system. To this end, we selected a wide range of conditions for testing, in part derived from literature and in part from previous findings obtained independently in our laboratories.

Two recent reports have demonstrated a preference of the parasite for young reticulocyte [25, 26]. The reticulocyte-prone rodent parasite *P*. *yoelii*, which mimics several biological features of *P*. *vivax*, has a strong tropism for the immature reticulocytes present in the bone marrow and spleen of BALB/c mice [25]. A second study found that clinical isolates of *P*. *vivax* from Thailand exhibited a strong preference for very young reticulocytes (CD71^high^ and CD71^med^) *in vitro* [26]. Therefore, to confirm these two studies, we looked at the parasites coming directly out of the *Aotus* monkey (*ex vivo*). Like the rodent study, we observed that the majority of rings were found in young reticulocytes Heilmeyer I and II-III (Fig 2). Interestingly, most of the *ex-vivo* trophozoites were also found inside reticulum-positive reticulocytes (Heilmeyer II-III and Heilmeyer IV Fig 2). This is in contrast to Malleret et al. who have reported that invasion would trigger an accelerated maturation of the reticulocyte such that reticulum would be expected to be absent by the trophozoite stage [26]. We did observe, however, that the *in vitro* rings and trophozoites (second generation) were found in older reticulocytes and mature RBCs, which is consistent with [26]. Whether *the ex* vivo *and in vitro* discrepancies are linked either to an intrinsic difference between the non-human primate adapted strain *P*. *vivax* Sal-1 and the Thai isolates, or to an *ex vivo* versus *in vitro* effect on the infected host cells needs to be further explored. Alternatively, the reticulocytes may mature *in vitro* prior to invasion, which could explain why rings are found in older cells. More detailed studies are needed to fully confirm these differences but the overall conclusion that we can draw from our findings is that *P*. *vivax* rings *ex vivo* are found in young reticulocytes indicating a preference for invasion of the youngest reticulocytes.

As reticulocytes represent only a minor portion of whole blood samples [27], enrichment is necessary to increase the numbers of susceptible host cells for invasion *in vitro*. We wondered if reticulocyte preparation methods could affect the parasite invasion as specific enrichment procedures (e.g. Percoll) may alter the make up of surface cell markers thereby negatively influencing parasite invasion [13]. Therefore, we opted for a head-to-head comparison of three different enrichment methodologies: differential centrifugation, Percoll enrichment (density gradient) and a CD71^+^ immuno-magnetic purification method Interestingly, we found that the reticulocytes enriched by density centrifugation alone could support better invasion of the parasites suggesting that at least Percoll density gradients may negatively impact the cells overall (Fig 3). Another density gradient method [20] using Nycodenz was published after the conclusion of this study and was therefore not tested during the studies presented in this manuscript. While Percoll is made up of colloidal silica coated with polyvinylpyrrolidone, which can be toxic to cells, Nycodenz is a nonionic iodinated gradient medium, which is nontoxic. It has been shown that Percoll gradients can damage the membranes of sperm [28]. However, no side-by-side comparisons were made in [20], which would be important to consider. Taken together, however, these experiments and the report published by Roobsoong et al. [20], demonstrate that the host cell health plays an important role in the outcome of *in vitro* cultures.

In the context of differential centrifugation, it is interesting to note that Golenda et al. [14] utilized a laborious method in which they achieved a five-fold increase in the number of reticulocytes (from 3–5% to 15–20%) after a subset of centrifugation and incubation steps with homologous serum. We used both the protocol described in Golenda et al. [14] as well as a simplified version with centrifugations at a maximum of 3,000 *xg* instead of the 35,000 *xg* ultra-centrifugations. With both procedures, we could obtain a maximum 3-fold increase in reticulocytes compared to the starting hemochromatosis blood reticulocytemia. A contributing factor in the difference in enrichment outcomes (5-fold reported by Golenda et al. [14] and 3-fold obtained in our laboratories) may be due to different treatment procedures for patients suffering from hemochromatosis today compared to 1997 [29]. In the past, repeated bleeds were performed prior to phlebotomy, resulting in an induction of reticulocytosis, while today phlebotomies are performed directly. Thus, the reticulocyte content in blood collected in the past was higher than the reticulocytes obtained from hemochromatosis patients today. Our data suggest that a lower number of reticulocytes in the blood at the start of the differential centrifugation procedures resulted in lower enrichment rates. Additionally, the repeat phlebotomies may also have resulted in a larger pool of young reticulocytes (post-emergence from the bone marrow) which may have resulted in higher invasion efficiencies and allowed for the doubling of the parasites at each generation in the Golenda et al. study [14]. This is also consistent with the preference we observed *ex vivo* with rings being found in the youngest reticulocytes.

Different factors may explain the difference in replication rates and parasitemia observed in our experiments compared to Golenda et al. [14]: (a) as explained above, reduced numbers of reticulocytes in the blood used to culture *P*. *vivax*, suggesting that a reticulocytes source other than the standardly prepared hemochromatosis blood is required if the 15–20% reticulocyte enrichment levels obtained by Golenda et al [14] through differential centrifugation, and reported to be instrumental to maintenance of good *P*. *vivax* multiplication rates and parasitemia rates, are to be achieved; (b) the different *P*. *vivax* parasite strain used (Chesson [14] vs Sal-1), suggesting that multiplication rates and parasitemia may be at least in part an intrinsic characteristics of the specific *P*. *vivax* strain, (c) the karyotype of *Aotus* monkey used (*nancymai* and *lemurinus griseimembra* [14] *vs lemurinus lemurinus)* and adaptations related to these parasite-host interactions may influence the adaptability of specific *P*. *vivax* strains to culture or the longer amount of time in which the parasite was hosted in the donor monkey, potentially allowing for *P*. *vivax* to stabilize and synchronize (17–21 days [14] vs 11–14 days).

Although the results of the Golenda study could not be replicated [14], we set out to compare a number of related variables: leukocyte depletion and its relation to the synchronicity of the starting parasite population; media conditions and supplements; and static versus dynamic culture conditions to improve culture conditions.

The choice of a leukocyte depletion method is important when setting up an *in vitro Plasmodium* culture as the presence of leukocytes is detrimental to parasite survival and the method chosen can influence the parasite population used to start the culture (parasitemia, stage, viability). In general, no consistent difference was observed in maturation rates when *P*. *vivax*-infected reticulocytes were purified from *Aotus* blood draws using either CF11-powder columns or Plasmodipur filters (Fig 4). However, the use of CF11 columns for leukocyte depletion from *Aotus* blood samples frequently resulted in the isolation of a mix-stage parasite population including young and mature parasite forms. In contrast, the use of Plasmodipur mostly resulted in the isolation of synchronous ring-stage parasite populations and much lower parasitemias. However, the CF11 parasites appeared to have reduced invasion efficiencies (Fig 4). Different hypotheses can be put forward to explain this finding: it is possible that a greater proportion of late stages are allowed through the CF11 column and these might be potentially less fit for adaptation to *in vitro* conditions. Alternatively, the shear forces involved in CF11 filtration may have a detrimental effect on the fitness of the late stages.

Along this same lines, starting parasite cultures using well-synchronized ring-stage populations [14] could improve progression of the culture as theoretically less metabolically active ring stages could better adapt to an *in vitro* environment while more mature stages, already exponentially increasing its metabolism, would suffer a “shock” with the sudden changes on the evolving environment. Conversely, the majority of *P*. *vivax in vitro* drug assays published to date rely on setting up cultures with samples rich in mature trophozoites [30, 31], which are allowed to develop for 20-24h to schizonts and then enriched by gradient techniques to overcome the reduction in parasitemia commonly observed during *in vitro* maturation. In our experiments we could not demonstrate the benefit from a 24 hr *in vitro* adaptation of rings stages to culture conditions as decreasing parasitemias were the constant and we could observe invasion in *P*. *vivax* cultures starting with either rings and or mixed stages.

The choice of an appropriate medium composition for parasite growth cannot be underestimated as, to complete the cycle, the parasite needs to take in nutrients from the media and not be overexposed to toxic by-products. In general, all McCoy’s 5A media contain L-glutamine, an essential amino acid for energy production as well as protein and nucleic acid synthesis. However, L-glutamine is quite unstable and is known to be subject to spontaneous degradation accompanied by the generation of byproducts such as ammonia and pyrrolidone carboxylic acid. GlutaMAX is a commercially available alternative to L-glutamine (dipeptide, L-alanine-L-glutamine), which is more stable and does not spontaneously degrade. The mechanism of dipeptide utilization by the parasite involves the gradual release of peptidase during the life cycle in culture, which allows for the gradual hydrolysis of the dipeptide in the medium resulting is an efficient energy metabolism and a high-growth yield. In our experiments, we found that adding GlutaMAX to the medium was beneficial for *P*. *vivax* maturation (Fig 5), although the parasitemia was not statically significant, we found a longer persistence of parasites *in vitro*, better conversion and second round invasion compared to the normal base media containing L-glutamine. This indicates that *P*. *vivax* may be sensitive to ammonia and pyrrolidone carboxylic acid.

Literature [12, 14] suggests that *P*. *vivax* may benefit from the alternation of static and shaking conditions as well as from an increase in hct (from 6 to 12% in Golenda et al [14]) just prior to reinvasion. In this context, Bass and Johnson 1912, suggested that invasion of reticulocytes by *P*. *vivax* should physiologically occur through direct contact between a fully multinucleated schizont and its target cell. This theory is supported by more recent data [32] showing that some of the *P*. *vivax* biomass is able to sequester in hematopoietic organs with slow circulation such as bone marrow and spleen (therefore facilitating cell-to-cell encounter) where the *P*. vivax parasite has access to an environment more rich in reticulocytes (especially the more immature subpopulations) than in peripheral blood. While based on this evidence it appears important to mimic a physiological template of constant circulation conditions through shaking, that would increase the chances of merozoites encountering reticulocytes, the time-point chosen for shifting from static to dynamic conditions may be critical. In fact, there is concern that changing from static to shaking conditions when *P*. *vivax* schizonts are already mature, could be detrimental on the *P*. *vivax in vitro* culture due to the fragility of this parasite stage [12]. Therefore, the shift from static to shaking conditions was performed early, prior to complete maturation of schizonts, in order to profit also from the early release of merozoite from early schizonts. Our data suggest that despite the marginal benefit shaking provides in terms of maturation, egress and re-invasion, this effect is not consistent across multiple samples and seems to depend largely on the staging of the starting population (Fig 6). Our findings suggest that shaking at 100rpm does not harm either maturation or invasion. Furthermore, we found that doubling the hct when the culture is first set up is beneficial to *P*. *vivax* growth (Fig 6). Although, in principle this appears counter-intuitive as a higher hct leads to a decrease in the medium nutrients available to the parasite, the beneficial effect observed may be explained by the need for a certain parasite/cell density to promote active communication between parasites through an exosome-like mechanism and/or a more direct contact among cells [33, 34]. In fact, we found that increased hct supported better parasite development (Fig 7), but this was due to an increase in uninfected cells rather than total parasite biomass.

In conclusion, while *Plasmodium falciparum* is the most life threatening of the 5 malaria parasite species-affecting humans, *P*. *vivax* causes the highest morbidity and is the most geographically widespread of the human *Plasmodium*. Therefore, there is a need to target this parasite in order to achieve malaria eradication. In order to make advances in the understanding of *P*. *vivax*’s biology, an *in vitro* culture for this parasite is urgently needed. Currently, research on *P*. *vivax* relies either on the use of field isolates or on the use of non-human primates adapted strains such as Sal-1, AMRU and Chesson [32]. Owing to their reproducibility, non-human primate samples have been widely used for *P*. *vivax* drug and vaccine *in vitro* and *in vivo* assays, and in the most successful attempt to date to establish an *in vitro* culture [14]. As we look to establish conditions to improve existing short-term *in vitro P*. *vivax* cultures and move towards establishing long-term cultures, we have chosen to use the well-adapted NHP *P*. *vivax* strain Sal-1 obtained from *Aotus lemurinus lemurinus* to benchmark parasite characteristics (specific media preferences, bottlenecks in *in vitro* growth and/or invasion). Although more experiments are needed to be performed some of the trends that were observed, our preliminary findings suggest that *P*. *vivax* Sal-1 strain invasion efficiency in culture was dependent on the reticulocyte enrichment method chosen and that parasite growth was positively influenced by the supplementation of culture media with stabilized nutrients indicating that *P*. *vivax* may be extremely sensitive to waste products. While the leuko-depletion method used appeared to have little impact on parasite progress, preliminary evidence suggests that shaking conditions and haematocrit may affect both *P*. *vivax* invasion and growth. Although further experiments are warranted to further investigate the huge number of variables able to influence *P*. *vivax* invasion, growth and egress, many of them not attempted in this work, we believe that the systematic approach presented in this paper, represents a point of departure for investigators interested in establishing long-term *P*. *vivax* cultures both with isolates from people in endemic countries and non-human primate samples.

## Supporting Information

S1 Fig *Ex vivo P*. *vivax* Sal-1 is found in reticulocytes of different maturity. (**A**) Heilmeyer classification according to [10,19]. (**B**- **E**) New methylene blue (NMB) and Giemsa stained smears of *P*. *vivax* Sal-1 rings after collection found in: non-NMB stained cell (erythrocyte) next to a non infected NMB stained reticulocyte (**B**), NMB weakly-stained reticulocytes (Heilmeyer IV) (**C**), NMB moderately-stained reticulocytes (Heilmeyer III-II) (**D**), NMB strongly-stained reticulocytes (Heilmeyer I) (**E**). (**F-G**) NMB and Giemsa stained smears of *P*. *vivax* Sal-1 mature stages after collection found in: NMB weakly-stained reticulocytes (Heilmeyer IV) (**F**), NMB moderately-stained reticulocytes (Heilmeyer III-II) (**G**). (**H-I**) Giemsa stained smears of *P*. *vivax* Sal-1 2^nd^ generation parasites after 20hr *in vitro* showing persistence of reticulum network (H and I corresponding to Heilmeyer IV and III-II respectively). Cells that do not contain NMB staining are considered mature RBCs.
(PNG)

S2 Fig Reticulocyte enrichment method can affect invasion of *P*. *vivax* Sal-1. (**A**) Flow cytometry measurements comparing the invasion of *P*. *vivax Sal-1* into human (GYPA+) reticulocytes obtained from different sources (Hemochromatosis vs Buffy coat) and with different enrichment methods (Percoll, density and beads) vs Aotus (GYPA-) reticulocytes. Analysis was performed on a Guava EasyCyte from Milipore flow cytometer at different time points from 0 to 72h after color compensation with beads following the manufacture’s protocol. Briefly, over 1x10^6^ cells from culture plates were washed twice with 200uL PBS-0.05% BSA, fixed with 100uL of 0.05% glutharaldehide for 15 min at 4°C, washed again with 200uL PBS-0.05% BSA, permeabilized with 100uL of 0.3% Triton X-100 for 5 min at RT and washed again with 200uL PBS-0.05% BSA. The pellet was re-suspended with 100uL PBS-0.05% BSA and stained with PyBiP-alexa647 (1:100 dilution), human GYPA (CD235a)-FITC (1:20 dilution) and CD71-PE (1:50 dilution). >1x10^5^ events were counted and recorded. Data was analysed using the FlowJo. Flow cytometry demonstrates the majority of the invasion events occurred in human cells (white bars) regardless of the reticulocyte source and preparation method. (**B**) PEMR of invasion using frozen reticulocytes from hemochromatosis blood enriched either by density or Percoll compared to fresh reticulocytes from hemochromatosis blood enriched by the same methods. The amount of cells recovered post-thaw limited the invasion assay to a single assay with MN28014. The fresh cells from MN28014 are shown. (**C**) Corresponding ring parasitemias from B. While a trend is observed, it was not possible to do statics with a single replicate.
(PNG)

S3 Fig Starting haematocrit (hct) can influence *in vitro* outcome of *P*. *vivax* Sal-1. For one biological replicate, MN26023a, the static culture was initiated at different hematocrits (hct), 6% or 15% with blood from the draw. The graph shows the difference in parasitemia during the *in vitro* culture.
(TIFF)

## References

[1] WHO World Malaria Report 2015

[2] Baird J. Evidence and implications of mortality associated with acute *Plasmodium vivax* Malaria. Clin Microbiol Rev. 2013;26(1):36–57. 10.1128/cmr.00074-12 23297258 PMC3553673

[3] Lacerda M, Fragoso S, Alecrim M Alexandre MA, Magalhães BM, Siqueira AM, et al Postmortem characterization of patients with clinical diagnosis of *Plasmodium vivax* malaria: To what extent does this parasite kill?. Clin Infect Dis. 2012;55(8):e67–e74. 10.1093/cid/cis615 22772803

[4] Battle KE, Gething PW, Elyazar IR, Moyes CL, Sinka ME, Howes RE, et al The global public health significance of Plasmodium vivax. Adv Parasitol. 2012;80:1–111 10.1016/B978-0-12-397900-1.00001-3 23199486

[5] Bousema T, Drakeley C. Epidemiology and infectivity of *Plasmodium falciparum* and *Plasmodium vivax* gametocytes in relation to malaria control and elimination. Clin Microbiol Rev. 2011;24(2):377–410. 10.1128/cmr.00051-10 21482730 PMC3122489

[6] Mons B. Preferential invasion of malarial merozoites into young red blood cells. Blood Cells. 1990;16(2–3):299–312. 2257316

[7] Sandberg S, Rustad P, Johannesen B, Stølsnes B. Within-subject biological variation of reticulocytes and reticulocyte-derived parameters. Eur J Haematol. 1998 7;61(1):42–8. 9688291 10.1111/j.1600-0609.1998.tb01059.x

[8] Neafsey D, Galinsky K, Jiang R Young L, Sykes SM, Saif S, et al The malaria parasite *Plasmodium vivax* exhibits greater genetic diversity than *Plasmodium falciparum*. Nat Genet. 2012;44(9):1046–1050. 10.1038/ng.2373 22863733 PMC3432710

[9] Lee E, Choi H, Hwang J, Hoh J, Cho Y, Baek E. The RNA in reticulocytes is not just debris: It is necessary for the final stages of erythrocyte formation. Blood Cells Mol Dis. 2014;53(1–2):1–10. 10.1016/j.bcmd.2014.02.009 24594313

[10] Malleret B, Xu F, Mohandas N, Suwanarusk R, Chu C, Leite JA, et al Significant biochemical, biophysical and metabolic diversity in circulating human cord blood reticulocytes. PLoS One. 2013 10 8;8(10) 10.1371/journal.pone.0076062 PMC3793000 24116088

[11] Chotivanich K, Silamut K, Udomsangpetch R, Stepniewska KA, Pukrittayakamee S, Looareesuwan S, et al Ex-vivo short-term culture and developmental assessment of Plasmodium vivax. Trans R Soc Trop Med Hyg. 2001;95(6):677–680. 10.1016/s0035-9203(01)90113-0 11816444

[12] Mons B, Collins W, Skinner J, van der Star W, Croon J, van der Kaay H. *Plasmodium vivax*: *In vitro* growth and reinvasion in red blood cells of *Aotus nancymai*. Experimental Parasitology. 1988;66(2):183–188. 10.1016/0014-4894(88)90089-6 3294025

[13] Lanners H. Prolonged *in vitro* cultivation of *Plasmodium vivax* using Trager's continuous-flow method. Parasitol Res. 1992;78(8):699–701. 10.1007/bf00931524 1480609

[14] Golenda C, Li J, Rosenberg R. Continuous *in vitro* propagation of the malaria parasite *Plasmodium vivax*. Proc Natl Acad Sci U S A. 1997;94(13):6786–6791. 10.1073/pnas.94.13.6786 9192643 PMC21236

[15] Methods in Malaria Research 2004.

[16] Obaldia N III, Otero W, Marin C, Aparicio J, Cisneros G. Long-term effect of a simple nest-box on the reproductive efficiency and other life traits of an *Aotus lemurinus lemurinus* monkey colony: an animal model for malaria research. J Med Primatol. 2011;40(6):383–391. 10.1111/j.1600-0684.2011.00489.x 21781134

[17] Bowers K, Bell D, Chiodini P, Barnwell J, Incardona S, Yen S, et al Inter-rater reliability of malaria parasite counts and comparison of methods. Malar J. 2009;8(1):267 10.1186/1475-2875-8-267 19939271 PMC2789092

[18] Lim C, Hansen E, DeSimone T, Moreno Y, Junker K, Bei A, et al Expansion of host cellular niche can drive adaptation of a zoonotic malaria parasite to humans. Nat Commun. 2013;4:1638 10.1038/ncomms2612 23535659 PMC3762474

[19] Heilmeyer L, Westha üser R. Reifungsstadien an U$\ddot{b}$erlebenden Reticu- lozyten In Vitro und ihre Bedeutung für die Schaetzung der täglichen Haemoglobin-Produktion *In Vivo*. Ztschr klin *Med*. 1932;121: 361–379.

[20] Roobsoong W, Tharinjaroen C, Rachaphaew N, Chobson P, Schofield L, Cui L, et al Improvement of culture conditions for long-term in vitro culture of *Plasmodium vivax*. Malar J. 2015;14(1). 10.1186/s12936-015-0815-z PMC4524445 26243280

[21] Roobsoong W, Maher S, Rachaphaew N, Barnes SJ, Williamson KC, Sattabongkot J, et al A rapid sensitive, flow cytometry-based method for the detection of *Plasmodium vivax*-infected blood cells. Malar J. 2014;13(1):55 10.1186/1475-2875-13-55 24528780 PMC3942109

[22] Bass C. The cultivation of malarial Plasmodia (*Plasmodium vivax* and *Plasmodium falciparum*) *in vitro*. J Exp Med. 1912;16(4):567–579. 10.1084/jem.16.4.567 19867597 PMC2124976

[23] Noulin F, Borlon C, Van Den Abbeele J, De Alessandro U, Erhart A. 1912–2012: a century of research on *Plasmodium vivax in vitro* culture. Trends in Parasitol. 2013;29(6):286–294. 10.1016/j.pt.2013.03.012 23623759

[24] Panichakul T, Sattabongkot J, Chotivanich K, Sirichaisinthop J, Cui L, Udomsangpetch R. Production of erythropoietic cells in vitro for continuous culture of *Plasmodium vivax*. Internat J Parasitol. 2007;37(14):1551–1557. 10.1016/j.ijpara.2007.05.009 17610880

[25] Martin-Jaular L, Elizalde-Torrent A, Thomson-Luque R, Ferrer M, Segovia JC, Herreros-Aviles E, et al Reticulocyte-prone malaria parasites predominantly invade CD71hi immature cells: implications for the development of an in vitro culture for *Plasmodium vivax*. Malar J. 2013;12(1):434 10.1186/1475-2875-12-434 24289105 PMC4220676

[26] Malleret B, Li A, Zhang R, Tan KS, Suwanarusk R, Claser C, et al Plasmodium vivax: restricted tropism and rapid remodeling of CD71-positive reticulocytes. *Blood*. 2014;125(8):1314–1324. 10.1182/blood-2014-08-596015 25414440 PMC4401350

[27] Mueller I, Galinski M, Baird J, Carlton JM, Kochar DK, Alonso PL, et al Key gaps in the knowledge of *Plasmodium vivax*, a neglected human malaria parasite. Lancet Infect Dis. 2009;9(9):555–566. 10.1016/s1473-3099(09)70177-x 19695492

[28] Oliveria LZ, Hossepian De Lima VFM, Levenhagen MA, Dos Santo RM, Assumpção TI, Jacomini JO, et al Transmission electron microscopy for characterization of acrosomal damage after Percoll gradient centrifugation of cryopreserved bovine spermatozoa. J. Vet. Sci. 2011;12(3)267–272. 21897100 10.4142/jvs.2011.12.3.267 PMC3165156

[29] Salgia R, Brown K. Diagnosis and management of hereditary hemochromatosis. Clin Liver Dis. 2015;19(1):187–198. 10.1016/j.cld.2014.09.011 25454304

[30] Russell B, Suwanarusk R, Borlon C, Costa FT, Chu CS, Rijken MJ, et al A reliable *ex vivo* invasion assay of human reticulocytes by *Plasmodium vivax*. Blood. 2011;118(13):e74–e81. 10.1182/blood-2011-04-348748 21768300 PMC3438884

[31] Noulin F, Borlon C, van den Eede P, Boel L, Verfaillie CM, D'Alessandro U, et al Cryopreserved reticulocytes derived from hematopoietic stem cells can be invaded by cryopreserved *Plasmodium vivax* isolates. PLoS ONE. 2012;7(7):e40798 10.1371/journal.pone.0040798 22844411 PMC3402485

[32] Obaldia N 3rd. Clinico-pathological observations on the pathogenesis of severe thrombocytopenia and anemia induced by *Plasmodium vivax* infections during antimalarial drug efficacy trials in *Aotus* monkeys. Am J Trop Med Hyg. 2007;77(1):3–13. Epub 2007/07/11. . 17620623

[33] Mantel P-Y, Hoang AN, Goldowitz I, Potashnikova D, Hamza B, Vorobjev I, et al Malaria-infected erythrocyte-derived microvesicles mediate cellular communication within the parasite population and with the host immune system. Cell Host Microbe 2013: 13(5), 521–34. 10.1016/j.chom.2013.04.009 23684304 PMC3687518

[34] Regev-Rudzki N, Wilson DW, Carvalho TG, Sisquella X, Coleman BM, Rug M, et al, Cell-cell communication between malaria-infected red blood cells via exosome-like vesicles. Cell. 2013: 153(5), 1120–33. 10.1016/j.cell.2013.04.029 23683579

