# Supplementary figures and images for: Insights into an Optimization of Plasmodium vivax Sal-1 In Vitro Culture: The Aotus Primate Model
Source: PLoS Negl Trop Dis. 2016 Jul 27;10(7):e0004870. doi: 10.1371/journal.pntd.0004870 (PMC4963040; doi:10.1371/journal.pntd.0004870)

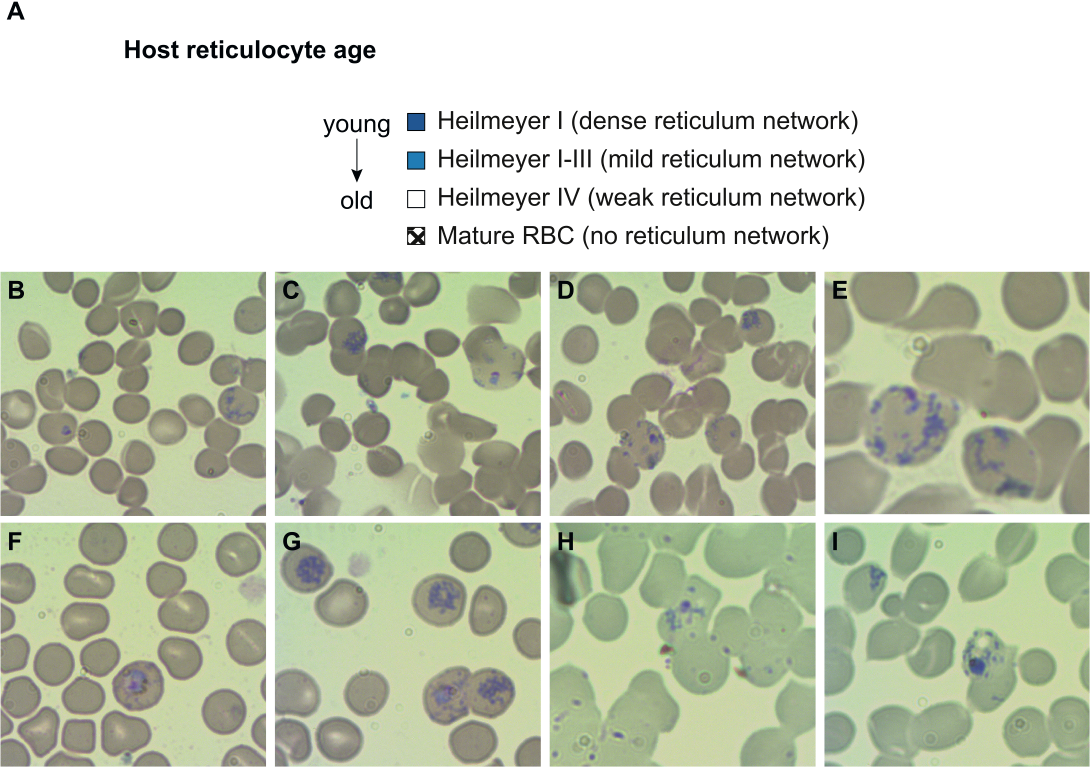

Supplement: S1 Fig — (A) Heilmeyer classification according to [10,19]. (B- E) New methylene blue (NMB) and Giemsa stained smears of P. vivax Sal-1 rings after collection found in: non-NMB stained cell (erythrocyte) next to a non infected NMB stained reticulocyte (B), NMB weakly-stained reticulocytes (Heilmeyer IV) (C), NMB moderately-stained reticulocytes (Heilmeyer III-II) (D), NMB strongly-stained reticulocytes (Heilmeyer I) (E). (F-G) NMB and Giemsa stained smears of P. vivax Sal-1 mature stages after collection found in: NMB weakly-stained reticulocytes (Heilmeyer IV) (F), NMB moderately-stained reticulocytes (Heilmeyer III-II) (G). (H-I) Giemsa stained smears of P. vivax Sal-1 2nd generation parasites after 20hr in vitro showing persistence of reticulum network (H and I corresponding to Heilmeyer IV and III-II respectively). Cells that do not contain NMB staining are considered mature RBCs. (PNG) [file pntd.0004870.s001.png]

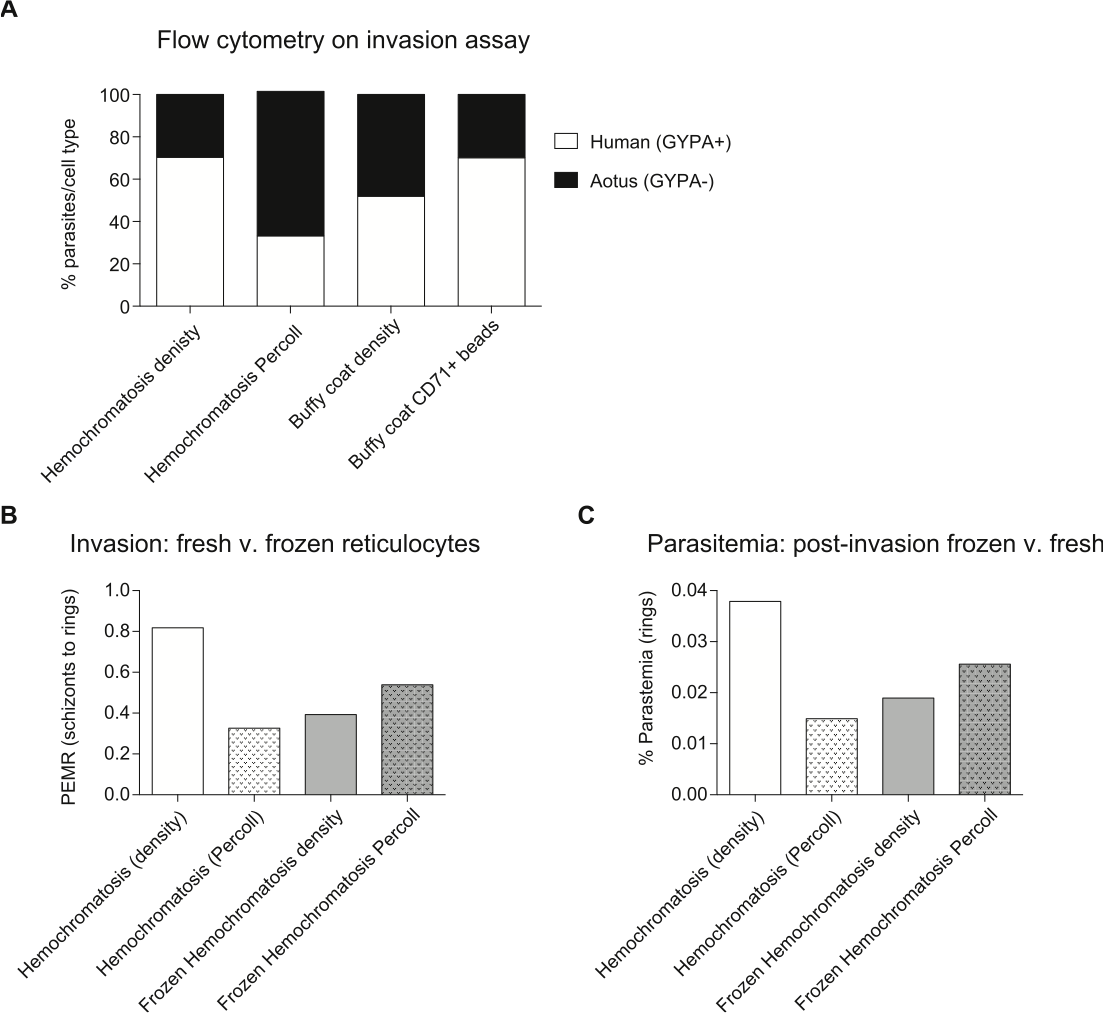

Supplement: S2 Fig — (A) Flow cytometry measurements comparing the invasion of P. vivax Sal-1 into human (GYPA+) reticulocytes obtained from different sources (Hemochromatosis vs Buffy coat) and with different enrichment methods (Percoll, density and beads) vs Aotus (GYPA-) reticulocytes. Analysis was performed on a Guava EasyCyte from Milipore flow cytometer at different time points from 0 to 72h after color compensation with beads following the manufacture’s protocol. Briefly, over 1x10^6^ cells from culture plates were washed twice with 200uL PBS-0.05% BSA, fixed with 100uL of 0.05% glutharaldehide for 15 min at 4°C, washed again with 200uL PBS-0.05% BSA, permeabilized with 100uL of 0.3% Triton X-100 for 5 min at RT and washed again with 200uL PBS-0.05% BSA. The pellet was re-suspended with 100uL PBS-0.05% BSA and stained with PyBiP-alexa647 (1:100 dilution), human GYPA (CD235a)-FITC (1:20 dilution) and CD71-PE (1:50 dilution). >1x10^5^ events were counted and recorded. Data was analysed using the FlowJo. Flow cytometry demonstrates the majority of the invasion events occurred in human cells (white bars) regardless of the reticulocyte source and preparation method. (B) PEMR of invasion using frozen reticulocytes from hemochromatosis blood enriched either by density or Percoll compared to fresh reticulocytes from hemochromatosis blood enriched by the same methods. The amount of cells recovered post-thaw limited the invasion assay to a single assay with MN28014. The fresh cells from MN28014 are shown. (C) Corresponding ring parasitemias from B. While a trend is observed, it was not possible to do statics with a single replicate. (PNG) [file pntd.0004870.s002.png]

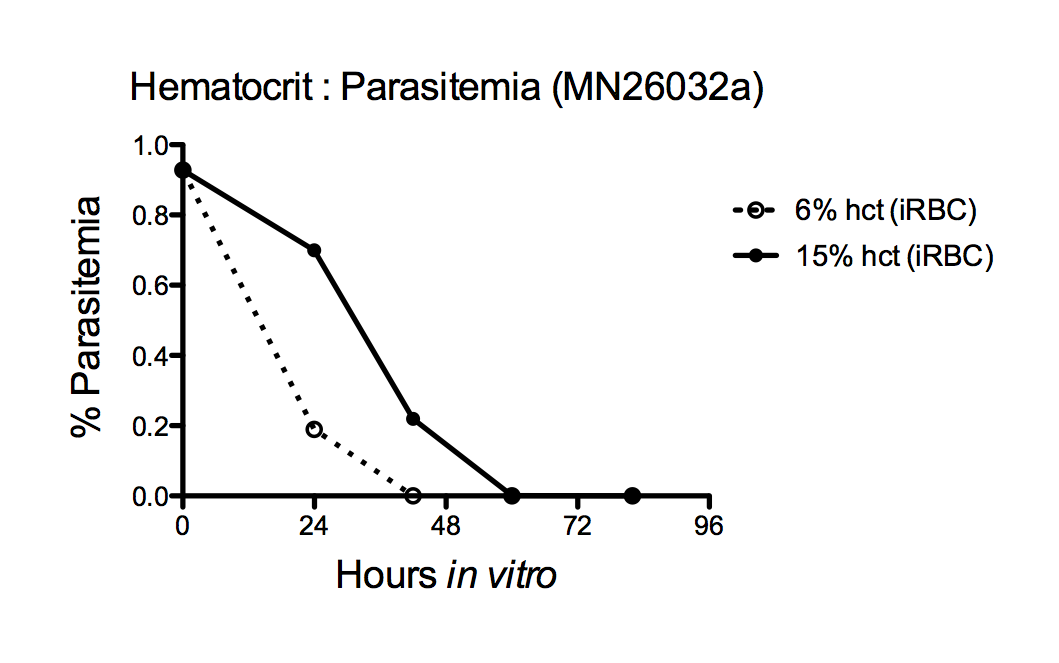

Supplement: S3 Fig — For one biological replicate, MN26023a, the static culture was initiated at different hematocrits (hct), 6% or 15% with blood from the draw. The graph shows the difference in parasitemia during the in vitro culture. (TIFF) [file pntd.0004870.s003.tiff]
